# Pediatric RSV-associated lower respiratory tract infection burden in G20 countries in 2023

**DOI:** 10.1016/j.isci.2026.117788

**Published:** 2026-10-07

**Authors:** Yan Guo, Yonghan Luo, Jie Zhang

**Affiliations:** 1 Department of Reproductive Gynecology, NHC Key Laboratory of Healthy Birth and Birth Defect Prevention in Western China First People’s Hospital of Yunnan Province, Kunming, Yunnan 650032, China; 2 Second Department of Infectious Disease, Kunming Children’s Hospital (Children’s Hospital Affiliated to Kunming Medical University), Kunming, Yunnan, China; 3 Department of Reproductive Gynecology, The Affiliated Hospital of Kunming University of Science and Technology, Kunming, Yunnan 650032, China; 4 Faculty of Life Science and Technology, Kunming University of Science and Technology, Kunming, Yunnan 650500, China

**Keywords:** respiratory syncytial virus, children, disease burden, global burden of disease, G20

## Abstract

Respiratory syncytial virus (RSV) is a leading cause of childhood lower respiratory infections, but updated G20 burden estimates are lacking. Using the Global Burden of Disease 2023 database, we analyzed deaths and disability-adjusted life years (DALYs) for RSV-associated lower respiratory infections in children aged 0–14 years across G20 members from 1990 to 2023, with stratification by sex, age, and country. In 2023, an estimated 5,373 deaths and 483,162 DALYs occurred, with mortality and DALY rates having declined by ∼5.5% annually since 1990. The COVID-19 pandemic drove burden to a historic low in 2021, but a marked rebound followed in 2022–2023, though rates remained below pre-pandemic levels. Infants aged <1 year bore the heaviest burden (mortality 8.688 per 100,000), and India ranked highest, whereas Australia ranked lowest. Burden strongly correlated inversely with Sociodemographic Index. These findings highlight the need for precision prevention, targeting the youngest children and lower-resource G20 members.

## Highlights

• Pediatric RSV burden in G20 fell sharply from 1990 to 2023
• COVID-19 drove RSV to a historic low in 2021, with a rebound in 2022–2023
• Infants under 1 year bore the heaviest RSV burden across G20 countries
• Lower SDI was strongly linked to higher pediatric RSV burden

## Graphical abstract

**Figure:**
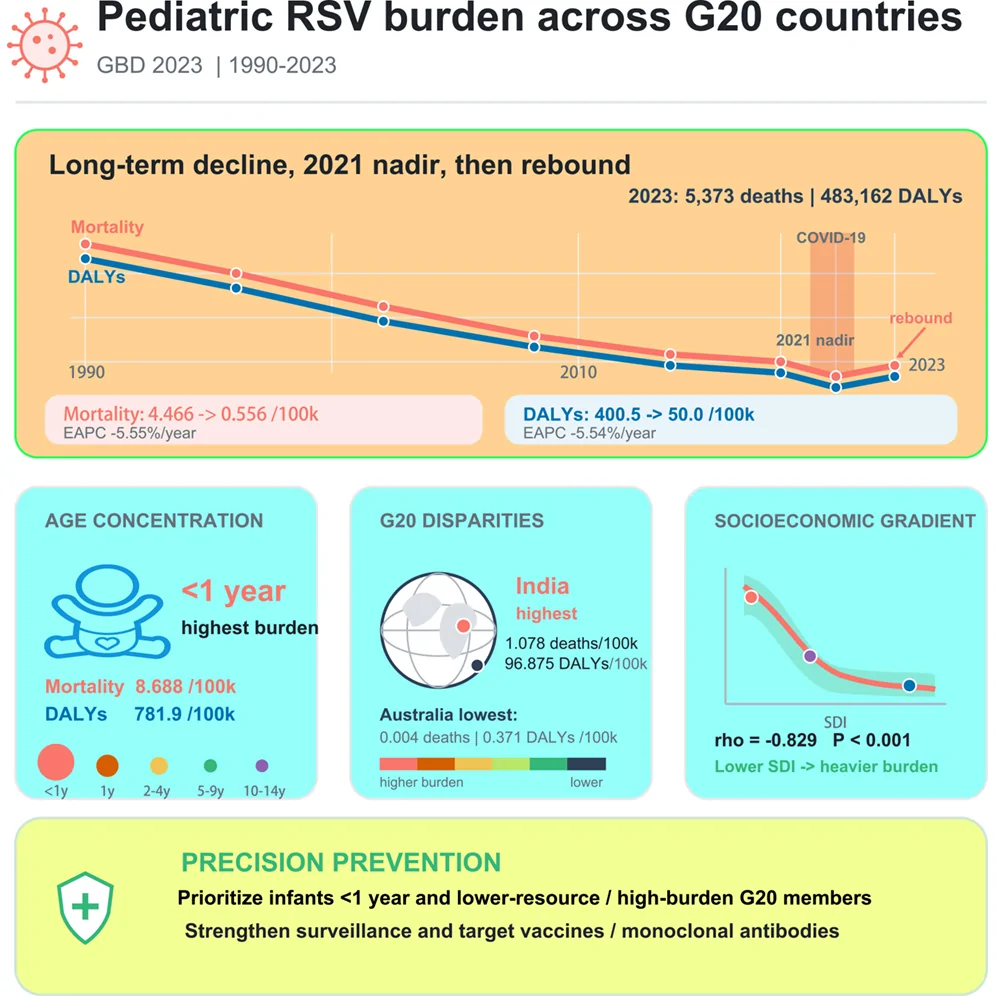

## Teaser

Health sciences; Medicine; Medical specialty; Medical microbiology; Public health

## Introduction

Respiratory syncytial virus (RSV) is a leading cause of acute lower respiratory tract infections among children worldwide. Belonging to the *Pneumoviridae* family, RSV is highly contagious and primarily transmitted via respiratory droplets and direct contact. In temperate regions, seasonal epidemics typically peak during winter and early spring. Although RSV can infect individuals across all age groups, infants, older adults, and immunocompromised populations are most susceptible to severe disease. Most children experience at least one RSV infection by the age of 2 years, with the primary infection often being the most severe, manifesting as clinical presentations ranging from mild upper respiratory symptoms to severe bronchiolitis, pneumonia, and even respiratory failure.^1^^,^^2^^,^^3^

Globally, the epidemiological characteristics, seasonal patterns, and disease burden of RSV have remained intensive research foci. Previous investigations have primarily concentrated on age distribution and disease severity.^4^^,^^5^^,^^6^^,^^7^ Nevertheless, existing studies on RSV disease burden exhibit substantial limitations. Most analyses include data only up to 2021 or earlier, thus failing to capture the dramatic epidemiological shifts during the post-pandemic era.^8^^,^^9^ Non-pharmaceutical interventions implemented during the COVID-19 pandemic drove RSV transmission to historic lows; however, following the relaxation of containment measures, many countries and regions witnessed a vigorous resurgence in 2022–2023, accompanied by altered seasonal patterns and increased clinical severity.^4^^,^^7^^,^^10^

Furthermore, the Global Burden of Disease Study (GBD), led by the Institute for Health Metrics and Evaluation (IHME) at the University of Washington, systematically quantifies mortality, disability-adjusted life years (DALYs), and other burden metrics across diverse countries, regions, ages, sexes, and years, thereby providing a scientific foundation for global health policy-making.^11^ Although the GBD has regularly published estimates of RSV-associated lower respiratory tract infections, its analytical population encompasses all age groups and does not specifically focus on the pediatric population, which bears the most severe RSV consequences.^12^ Previous studies, such as Zhao et al.^13^ utilized GBD 1990–2021 data to describe the all-age RSV burden, reporting 31,525 deaths and 2.59 million DALYs globally in 2021, with the burden concentrated predominantly in South Asia and sub-Saharan Africa^8^; Lian et al.^14^ further revealed an age-polarized shift of RSV burden toward older adults.^9^ However, none of these studies provided updated global pediatric RSV burden estimates covering the peak pandemic period and subsequent years (2022–2023), a critical time frame during which RSV epidemiological patterns likely underwent profound changes.

The G20 (Group of Twenty) comprises 19 countries and the European Union, representing roughly two-thirds of the world’s population and more than 80% of global GDP. These nations encompass a wide spectrum of economic development, health system capacities, and epidemiological profiles, making them a crucial focus for understanding RSV burden and guiding policy. Therefore, this study leveraged the most recent GBD 2023 database to systematically evaluate the burden of RSV-associated lower respiratory tract infections in children within G20 countries from 1990 to 2023. We describe the global and regional mortality landscape in 2023; analyze temporal trends from 1990 to 2023; delineate disparities across regions, countries, age groups, and sexes; explore the correlation between the Sociodemographic Index (SDI) and disease burden. The findings aim to provide evidence-based support for optimizing pediatric RSV prevention and control strategies in the post-pandemic era, as well as for the precise deployment of vaccines and monoclonal antibodies.

## Results

### Disease burden of pediatric RSV-associated lower respiratory tract infections in G20 in 2023

In 2023, the estimated number of deaths due to pediatric RSV-associated lower respiratory tract infections in G20 countries was 5,373.225 (95% uncertainty interval [UI]: 3,715.553–7,862.622), corresponding to a mortality rate of 0.556 per 100,000 children (95% UI: 0.385–0.814). The DALYs were 483,161.884 (95% UI: 334,432.415–707,829.900), with a DALY rate of 50.020 per 100,000 (95% UI: 34.622–73.279). By sex, the mortality rate was 0.546 per 100,000 in males and 0.568 per 100,000 in females (Table 1) To further characterize the trajectory, we calculated segmented estimated annual percentage change (EAPC) for three distinct periods. The mortality rate declined with an EAPC of −4.06% (95% confidence interval [CI]: −4.17 to −3.94) during 1990–2019, followed by a sharp decline of −44.38% (95% CI: −98.37 to 1800.37) during 2019–2021, and then an increase with an EAPC of +13.54% (95% CI: −18.31 to 57.79) during 2021–2023. The DALY rate showed similar patterns, with EAPCs of −4.05% (95% CI: −4.17 to −3.94), −44.33% (95% CI: −98.37 to 1804.56), and +13.54% (95% CI: −18.65 to 58.47) across the three periods, respectively. These short-segment EAPC estimates are based on only three annual observations each and are presented as conditional point-estimate summaries; the wide CIs reflect substantial uncertainty and should be interpreted with caution. Formal UIs for descriptive rate changes are not reported, as these contrasts are derived from point estimates rather than matched draws.

**Table 1:** Deaths and disability-adjusted life years in G20 attributable to RSV-associated lower respiratory tract infections within G20 countries in children, 1990–2023

Level	1990 number (95% UI)	1990 rate per 100,000 (95% UI)	2023 number (95% UI)	2023 rate per 100,000 (95% UI)	Rate change	EAPC (95% CI)
Deaths-G20	45,619.852 (38,838.914–53,188.023)	4.466 (3.802–5.206)	5,373.225 (3,715.553–7,862.622)	0.556 (0.385–0.814)	−0.875 (−0.917 to −0.804)	−5.55 (−6.37 to −4.72)
Deaths-Argentina	67.976 (61.992–74.207)	0.667 (0.608–0.728)	4.378 (1.682–9.000)	0.043 (0.017–0.089)	−0.935 (−0.975 to −0.866)	−6.12 (−8.38 to −3.81)
Deaths-Australia	3.958 (3.582–4.484)	0.104 (0.095–0.118)	0.197 (0.089–0.340)	0.004 (0.002–0.007)	−0.961 (−0.983 to −0.929)	−6.23 (−9.55 to −2.78)
Deaths-Brazil	1,193.750 (1,057.141–1,348.250)	2.275 (2.015–2.570)	116.388 (55.308–208.382)	0.269 (0.128–0.481)	−0.882 (−0.944 to −0.792)	−6.27 (−7.34 to −5.19)
Deaths-Canada	6.271 (5.723–6.852)	0.108 (0.099–0.118)	3.213 (2.081–4.453)	0.052 (0.034–0.072)	−0.519 (−0.695 to −0.331)	−2.89 (−4.73 to −1.01)
Deaths-China	17,614.661 (14,921.723–20,468.140)	5.540 (4.693–6.437)	130.378 (71.719–202.861)	0.052 (0.028–0.080)	−0.991 (−0.995 to −0.985)	−13.00 (−14.69 to −11.28)
Deaths-United Kingdom	15.756 (14.250–17.508)	0.144 (0.130–0.160)	6.019 (4.583–8.131)	0.051 (0.039–0.069)	−0.645 (−0.725 to −0.522)	−3.93 (−5.80 to −2.01)
Deaths-France	8.856 (7.947–9.755)	0.075 (0.067–0.083)	4.425 (2.697–6.828)	0.036 (0.022–0.056)	−0.514 (−0.713 to −0.240)	−3.08 (−4.67 to −1.46)
Deaths-Germany	11.549 (10.604–12.800)	0.089 (0.082–0.099)	1.951 (1.326–2.818)	0.016 (0.011–0.024)	−0.817 (−0.882 to −0.732)	−4.61 (−7.27 to −1.88)
Deaths-India	21,296.403 (17,102.384–26,780.330)	6.675 (5.360–8.393)	3,908.194 (2,230.668–6,327.152)	1.078 (0.615–1.745)	−0.839 (−0.915 to −0.712)	−4.58 (−5.39 to −3.76)
Deaths-Indonesia	2,360.443 (1,582.950–3,321.965)	3.469 (2.327–4.883)	580.028 (291.696–954.189)	0.866 (0.435–1.425)	−0.750 (−0.887 to −0.535)	−5.68 (−7.55 to −3.76)
Deaths-Italy	11.007 (9.380–13.025)	0.119 (0.102–0.141)	2.446 (1.802–3.249)	0.033 (0.025–0.044)	−0.721 (−0.803 to −0.618)	−3.90 (−5.70 to −2.06)
Deaths-Japan	25.148 (22.260–28.933)	0.112 (0.099–0.128)	3.623 (2.109–5.599)	0.025 (0.015–0.039)	−0.776 (−0.870 to −0.647)	−5.96 (−9.63 to −2.14)
Deaths-Mexico	922.951 (832.208–1,032.342)	2.765 (2.493–3.093)	298.051 (204.084–410.774)	0.964 (0.660–1.328)	−0.651 (−0.768 to −0.526)	−4.18 (−5.61 to −2.72)
Deaths-Republic of Korea	35.288 (30.265–42.198)	0.308 (0.264–0.369)	0.787 (0.499–1.115)	0.014 (0.009–0.020)	−0.955 (−0.973 to −0.932)	−11.94 (−16.26 to −7.40)
Deaths-Russian Federation	458.491 (410.316–517.051)	1.313 (1.175–1.481)	49.119 (33.094–66.025)	0.213 (0.144–0.286)	−0.838 (−0.893 to −0.776)	−6.13 (−7.37 to −4.87)
Deaths-Saudi Arabia	111.425 (70.663–158.367)	1.607 (1.019–2.283)	62.446 (30.335–105.212)	0.775 (0.376–1.306)	−0.518 (−0.784 to −0.062)	−1.77 (−3.03 to −0.49)
Deaths-South Africa	469.497 (346.456–600.769)	3.423 (2.526–4.380)	76.092 (30.755–149.224)	0.473 (0.191–0.927)	−0.862 (−0.947 to −0.690)	−3.09 (−5.42 to −0.70)
Deaths-Türkiye	933.115 (622.607–1,264.264)	4.544 (3.032–6.156)	55.294 (27.983–91.802)	0.299 (0.151–0.496)	−0.934 (−0.971 to −0.873)	−8.04 (−9.16 to −6.90)
Deaths-United States of America	73.309 (65.700–81.829)	0.131 (0.117–0.146)	70.194 (49.111–90.272)	0.117 (0.082–0.151)	−0.104 (−0.394 to −0.204)	−0.63 (−2.05 to −0.81)
Deaths-European Union	347.486 (327.831–383.986)	0.420 (0.396–0.464)	52.353 (40.376–64.894)	0.078 (0.060–0.096)	−0.815 (−0.860 to −0.768)	−5.88 (−7.83 to −3.90)
Deaths-Male	24,465.859 (19,903.208–29,385.200)	4.631 (3.768–5.562)	2,742.604 (1,867.634–4,065.885)	0.546 (0.372–0.809)	−0.882 (−0.922 to −0.814)	−5.79 (−6.60 to −4.98)
Deaths-Female	21,153.993 (16,393.681–26,365.189)	4.288 (3.323–5.344)	2,630.621 (1,764.434–3,904.951)	0.568 (0.381–0.842)	−0.868 (−0.921 to −0.792)	−5.29 (−6.13 to −4.44)
Deaths-<1 year	36,725.464 (31,167.938–42,814.477)	52.659 (44.690–61.390)	4,484.103 (3,035.171–6,758.367)	8.688 (5.881–13.094)	−0.835(−0.893 to −0.743)	−5.18 (−5.87 to −4.48)
Deaths-12–23 months	3,622.754 (2,906.321–4,433.270)	5.226 (4.192–6.395)	389.683 (293.000–552.425)	0.732 (0.550–1.038)	−0.860 (−0.902 to −0.793)	−5.45 (−6.24 to −4.65)
Deaths-2–4 years	4,917.291 (3,859.424–6,076.510)	2.337 (1.834–2.888)	433.018 (322.297–617.731)	0.246 (0.183–0.351)	−0.895 (−0.926 to −0.842)	−6.34 (−7.22 to −5.45)
Deaths-5–9 years	246.774 (199.256–295.447)	0.071 (0.057–0.085)	36.108 (27.375–46.615)	0.011 (0.008–0.014)	−0.852 (−0.895 to −0.800)	−5.51 (−6.47 to −4.55)
Deaths-10–14 years	107.569 (88.575–130.302)	0.033 (0.027–0.040)	30.313 (23.026–40.395)	0.009 (0.007–0.012)	−0.733 (−0.813 to −0.631)	−3.62 (−4.36 to −2.87)
DALY-G20	4,091,464.436 (3,485,415.127–4,769,218.796)	400.498 (341.174–466.840)	483,161.884 (334,432.415–707,829.900)	50.020 (34.622–73.279)	−0.875 (−0.917–0.804)	−5.54 (−6.36 to −4.72)
DALY-Argentina	6,112.241 (5,568.023–6,659.820)	59.994 (54.652–65.368)	393.724 (151.592–807.626)	3.906 (1.504–8.012)	−0.935 (−0.975 to −0.866)	−6.11 (−8.37 to −3.80)
DALY-Australia	357.716 (323.127–404.677)	9.442 (8.529–10.682)	17.967 (8.210–30.945)	0.371 (0.169–0.639)	−0.961 (−0.983 to −0.928)	−6.22 (−9.55 to −2.78)
DALY-Brazil	108,697.245 (96,498.806–122,003.136)	207.184 (183.933–232.546)	10,492.359 (5,002.945–18,688.355)	24.206 (11.542–43.115)	−0.883 (−0.945 to −0.794)	−6.30 (−7.37 to −5.22)
DALY-Canada	563.743 (516.538–618.500)	9.725 (8.910–10.669)	289.449 (187.602–400.950)	4.686 (3.037–6.491)	−0.518 (−0.694 to −0.330)	−2.87 (−4.71 to −1.00)
DALY-China	1,582,868.354 (1,339,965.039–1839622.810)	497.826 (421.431–578.578)	12,039.571 (6,592.956–18,465.178)	4.776 (2.615–7.325)	−0.990 (−0.995 to −0.984)	−12.93 (−14.60 to −11.22)
DALY-United Kingdom	1,420.665 (1,285.474–1,571.573)	12.999 (11.762–14.380)	545.527 (417.449–735.204)	4.633 (3.546–6.244)	−0.644 (−0.723 to −0.523)	−3.90 (−5.77 to −1.99)
DALY-France	803.645 (724.889–886.671)	6.809 (6.142–7.512)	405.184 (244.599–618.758)	3.340 (2.016–5.100)	−0.509 (−0.712 to −0.234)	−3.03 (−4.62 to −1.41)
DALY-Germany	1,045.776 (962.226–1,162.591)	8.074 (7.429–8.976)	185.270 (125.478–265.274)	1.545 (1.047–2.213)	−0.809 (−0.875 to −0.725)	−4.51 (−7.15 to −1.80)
DALY-India	1,904,886.843 (1,530,497.159–2,394,199.339)	597.018 (479.679–750.375)	351,293.485 (200,755.870–568,712.054)	96.875 (55.362–156.832)	−0.838 (−0.914 to −0.711)	−4.57 (−5.38 to −3.75)
DALY-Indonesia	211,785.487 (142,671.637–297,486.936)	311.275 (209.694–437.236)	52,156.497 (26,307.863–85669.268)	77.865 (39.275–127.897)	−0.750 (−0.886 to −0.535)	−5.67 (−7.55 to −3.76)
DALY-Italy	992.216 (848.959–1,172.996)	10.753 (9.200–12.712)	223.349 (164.873–293.647)	3.040 (2.244–3.997)	−0.717 (−0.798 to −0.616)	−3.85 (−5.65 to −2.02)
DALY-Japan	2,280.859 (2,023.908–2,612.463)	10.121 (8.981–11.593)	335.552 (197.034–518.450)	2.316 (1.360–3.578)	−0.771 (−0.867 to −0.641)	−5.91 (−9.57 to −2.10)
DALY-Mexico	82,647.626 (74,530.229–92,387.029)	247.602 (223.283–276.780)	26,582.415 (18,204.785–36,629.842)	85.970 (58.876–118.464)	−0.653 (−0.768 to −0.528)	−4.19 (−5.62 to −2.74)
DALY-Republic of Korea	3,148.152 (2,716.098–3,745.870)	27.492 (23.719–32.712)	74.458 (47.778–106.103)	1.310 (0.841–1.867)	−0.952 (−0.971 to −0.929)	−11.76 (−16.08 to −7.22)
DALY-Russian Federation	41,136.690 (36,820.588–46,360.938)	117.844 (105.480–132.810)	4,412.833 (2,973.744–5,935.513)	19.148 (12.904–25.755)	−0.838 (−0.893 to −0.776)	−6.12 (−7.36 to −4.86)
DALY-Saudi Arabia	10,039.908 (6,427.266–14,169.449)	144.757 (92.669–204.297)	5,581.475 (2,709.389–9,390.747)	69.261 (33.621–116.530)	−0.522 (−0.785 to −0.069)	−1.80 (−3.05 to −0.53)
DALY-South Africa	42,143.826 (31,104.762–53,807.331)	307.264 (226.780–392.300)	6,818.674 (2,761.872–13,367.169)	42.370 (17.162–83.062)	−0.862 (−0.947 to −0.690)	−3.09 (−5.42 to −0.71)
DALY-Türkiye	83,916.984 (56,074.168–113,464.938)	408.612 (273.039–552.489)	4,997.132 (2,541.879–8,265.989)	27.022 (13.745–44.699)	−0.934 (−0.971 to −0.872)	−8.03 (−9.15 to −6.90)
DALY-United States of America	6,616.459 (5,955.603–7,373.304)	11.832 (10.650–13.185)	6,316.961 (4,402.445–8,102.637)	10.570 (7.367–13.558)	−0.107 (−0.396 to −0.197)	−0.65 (−2.07 to −0.78)
DALY-European Union	31,252.829 (29,486.716–34,498.893)	37.797 (35.661–41.723)	4,772.517 (3,683.776–5,907.908)	7.075 (5.461–8.758)	−0.813 (−0.857 to −0.765)	−5.83 (−7.77 to −3.85)
DALY-Male	2,197,192.581 (1,786,513.633–2,634,452.410)	415.919 (338.179–498.690)	246,819.301 (168,341.765–365443.891)	49.125 (33.505–72.735)	−0.882 (−0.922 to −0.814)	−5.79 (−6.59 to −4.98)
DALY-Female	1,894,271.855 (1,470,587.677–2,360,715.517)	383.984 (298.100–478.536)	236,342.583 (158,537.536–351,032.817)	50.990 (34.204–75.734)	−0.867 (−0.921 to −0.791)	−5.28 (−6.12 to −4.43)
DALY-<1 year	3,302,101.572 (2,802,204.005–3,847,717.037)	4,734.719 (4,017.941–5,517.050)	403,544.315 (273,294.222–607,975.118)	781.874 (529.512–1,177.962)	−0.835 (−0.893 to −0.743)	−5.18 (−5.87 to −4.47)
DALY-12–23 months	324,255.947 (259,923.833–396,013.640)	467.727 (374.930–571.234)	34,975.363 (26,324.008–49,401.240)	65.708 (49.455–92.810)	−0.860 (−0.901 to −0.793)	−5.44 (−6.23 to −4.64)
DALY-2-4 years	435,160.501 (342,062.434–536,627.869)	206.810 (162.565–255.032)	38,954.388 (28,943.693–55,658.891)	22.129 (16.442–31.619)	−0.893 (−0.924 to −0.840)	−6.30 (−7.18 to −5.42)
DALY-5-9 years	21,311.950 (17,217.842–25,341.558)	6.149 (4.968–7.312)	3,234.313 (2,472.247–4,115.587)	0.945 (0.723–1.203)	−0.846 (−0.891 to −0.794)	−5.40 (−6.34 to −4.45)
DALY-10–14 years	8,634.467 (7,163.352–10,370.799)	2.653 (2.201–3.186)	2,453.506 (1,877.616–3,214.980)	0.715 (0.547–0.937)	−0.730 (−0.811 to −0.628)	−3.59 (−4.33 to −2.84)

### Temporal trends in disease burden, 1990–2023

Between 1990 and 2023, the overall mortality rate for pediatric RSV-associated lower respiratory tract infections in G20 countries declined from 4.466 per 100,000 to 0.556 per 100,000, with an EAPC of −5.55% (95% CI: −6.37 to −4.72). The DALY rate decreased from 400.498 per 100,000 to 50.020 per 100,000. indicating a significant downward trend. Reductions were similar between males (EAPC: −5.79%) and females (EAPC: −5.28%) (Table 1; Figures 1A–1F).

**Figure 1:**
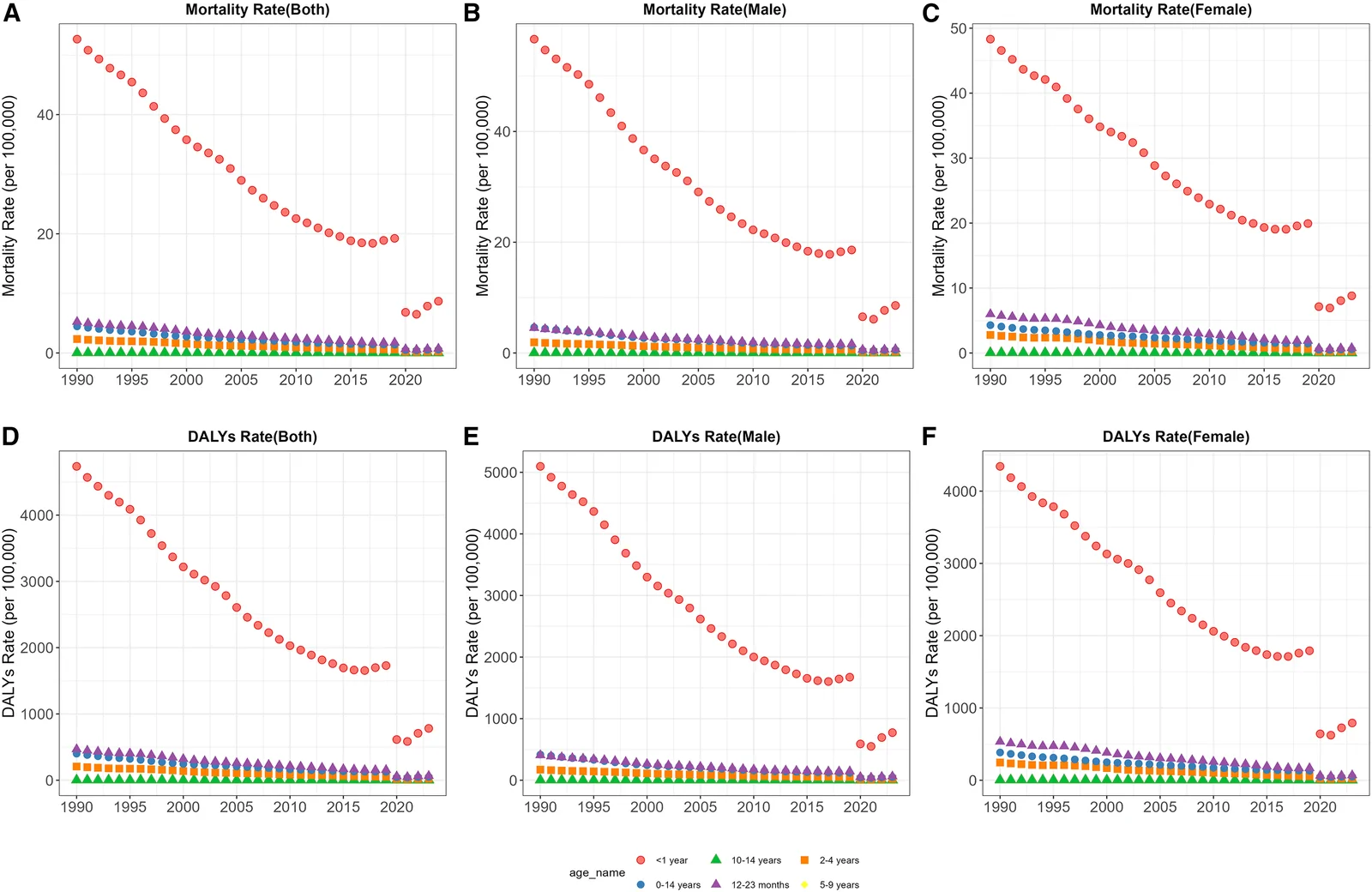
Temporal trends in the burden of RSV-associated lower respiratory tract infections among children aged 0–14 years in G20 countries by sex and age group, 1990–2023 (A–C) Mortality rates for both sexes combined, males, and females, respectively. (D–F) Disability-adjusted life year (DALY) rates for both sexes combined, males, and females, respectively.

Age-stratified analysis showed that infants aged <1 year had the highest burden (mortality rate 8.688 per 100,000; DALY rate 781.874 per 100,000), and this group also experienced a rapid decline (EAPC for mortality: −5.18%). Children aged 2–4 years showed the fastest decline (EAPC: −6.34%), while the 10–14 years group had the slowest (EAPC: −3.62%) (Table 1; Figures 1A–1F; Figures 2A and 2B).

**Figure 2:**
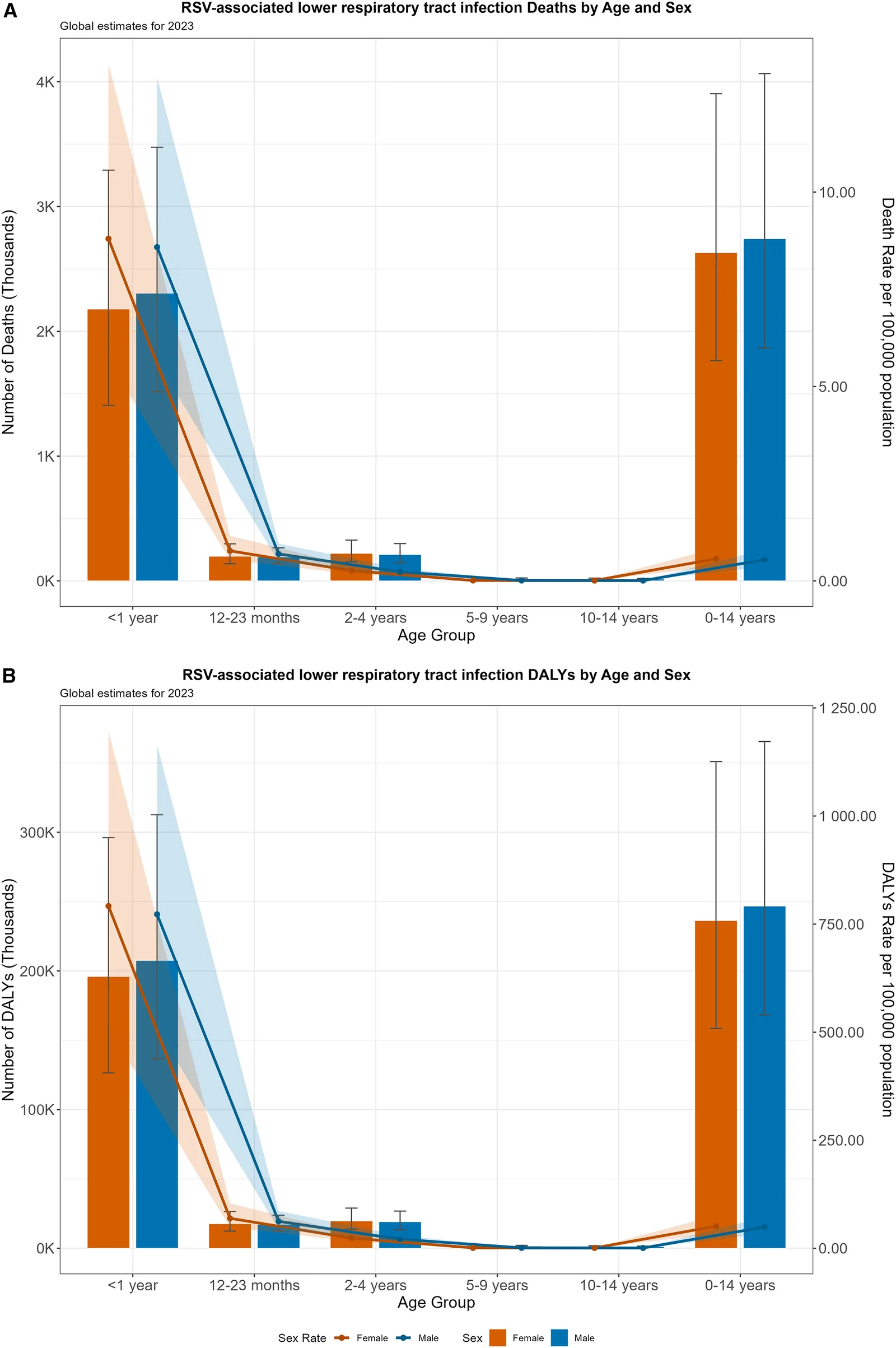
Distribution of deaths, mortality rates, and DALY burden due to RSV-associated lower respiratory tract infections in children aged 0–14 years in G20 countries by sex and age group, 2023 (A) Number of deaths and mortality rate by sex and age group. (B) Number of DALYs and DALY rate by sex and age group. Vertical error bars indicate 95% uncertainty intervals. Note: the “0–14 years” bars represent the aggregate total across all age subgroups, not a separate mutually exclusive age category.

### Disparities across G20 countries

Substantial heterogeneity existed across G20 member states in 2023 (Figures 3A and 3B). India recorded the highest number of deaths (3,908), followed by Indonesia (580) and China (130). In terms of mortality rate, India also ranked highest (1.078 per 100,000), followed by Mexico (0.964), Saudi Arabia (0.775), and South Africa (0.473). The lowest mortality rates were observed in Australia (0.004), the Republic of Korea (0.014), Germany (0.016), Japan (0.025), and United Kingdom (0.051).

**Figure 3:**
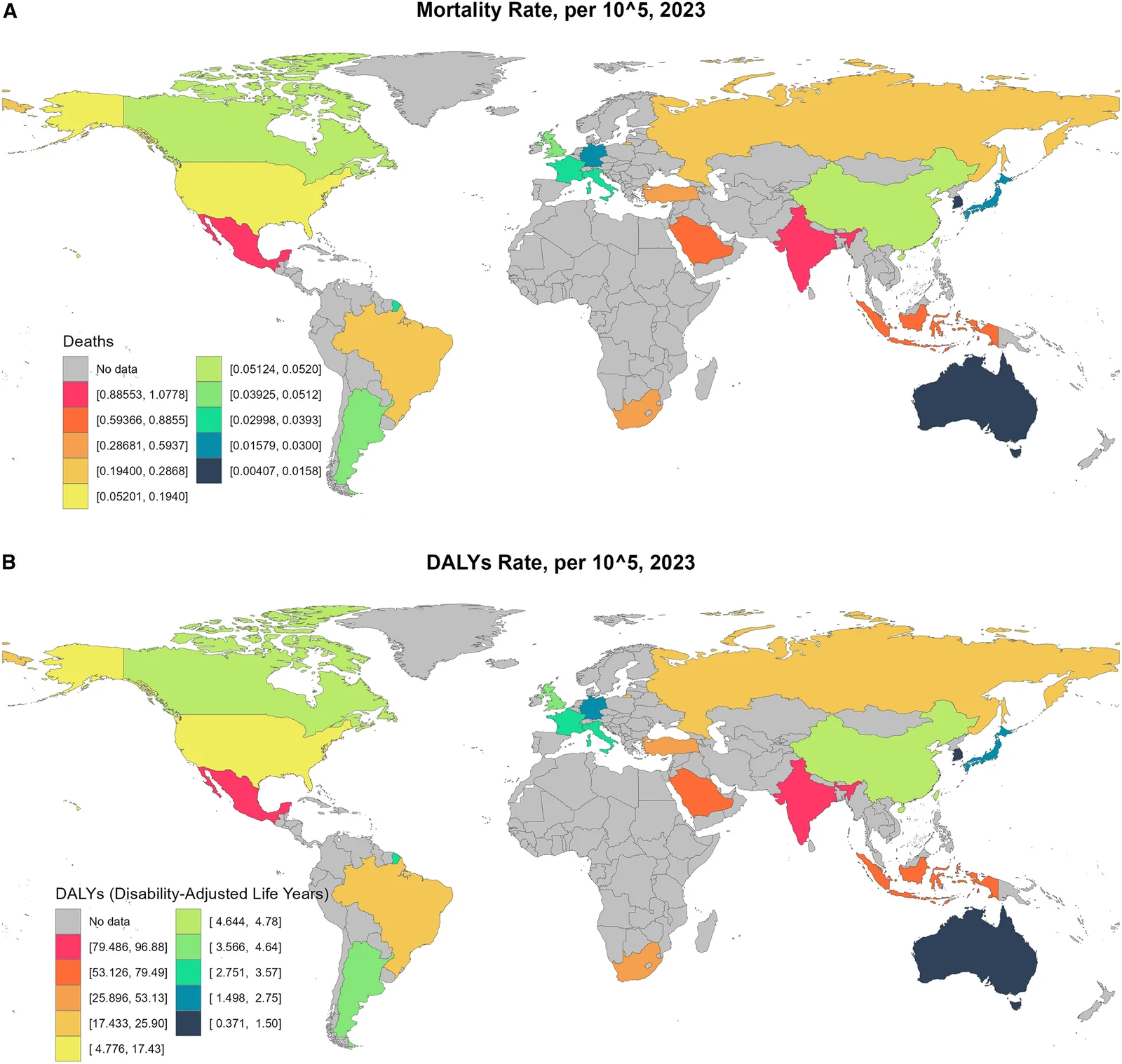
Geographic distribution of the burden of RSV-associated lower respiratory tract infections in children aged 0–14 years in G20 countries, 2023 (A) Mortality rate per 100,000 children by G20 member state. (B) DALY rate per 100,000 children by G20 member state.

The fastest declines in mortality rate from 1990 to 2023 occurred in China (EAPC: −13.00%), the Republic of Korea (−11.94%), and Turkey (−8.04%), while the United States showed a non-significant decline (EAPC: −0.63%), and Saudi Arabia had a relatively slow decrease (−1.77%). Other countries with large reductions included Argentina (−6.12%), Brazil (−6.27%), and Russia (−6.13%). The DALY rate patterns closely mirrored those of mortality: India had the highest DALY rate (96.875 per 100,000), followed by Mexico (85.970), Indonesia (77.865), and Saudi Arabia (69.261). The lowest DALY rates were in Australia (0.371), South Korea (1.310), Germany (1.545), and Japan (2.316) (see Table 1 for complete country-level data). To assess whether country rankings were influenced by differences in pediatric age composition, we performed age-stratified sensitivity analyses for each of the five age subgroups (<1 year, 1–2 years, 2–4 years, 5–9 years, and 10–14 years). The results (Table S1) showed that India, Indonesia, Mexico, Saudi Arabia, and South Africa consistently ranked among the highest-burden countries across nearly all age subgroups, while Australia, Germany, Japan, the Republic of Korea, and the United Kingdom consistently ranked among the lowest. This consistency supports the robustness of our country-level findings.

### Correlation between disease burden and the Sociodemographic Index

Spearman’s rank correlation analysis was performed at the national level to examine the association between SDI and both mortality and DALY rates among children aged 0–14 years in G20 countries. The results revealed a remarkably strong negative correlation between SDI and disease burden: the correlation coefficient for mortality was ρ = −0.829 (*p* < 0.001) and for DALY rate it was ρ = −0.829 (*p* < 0.001). These findings suggest a strong ecological association between lower socioeconomic development and a heavier RSV-related disease burden in children aged 0–14 years, although this association is descriptive at the national level and does not imply causality (Figures 4A and 4B).

**Figure 4:**
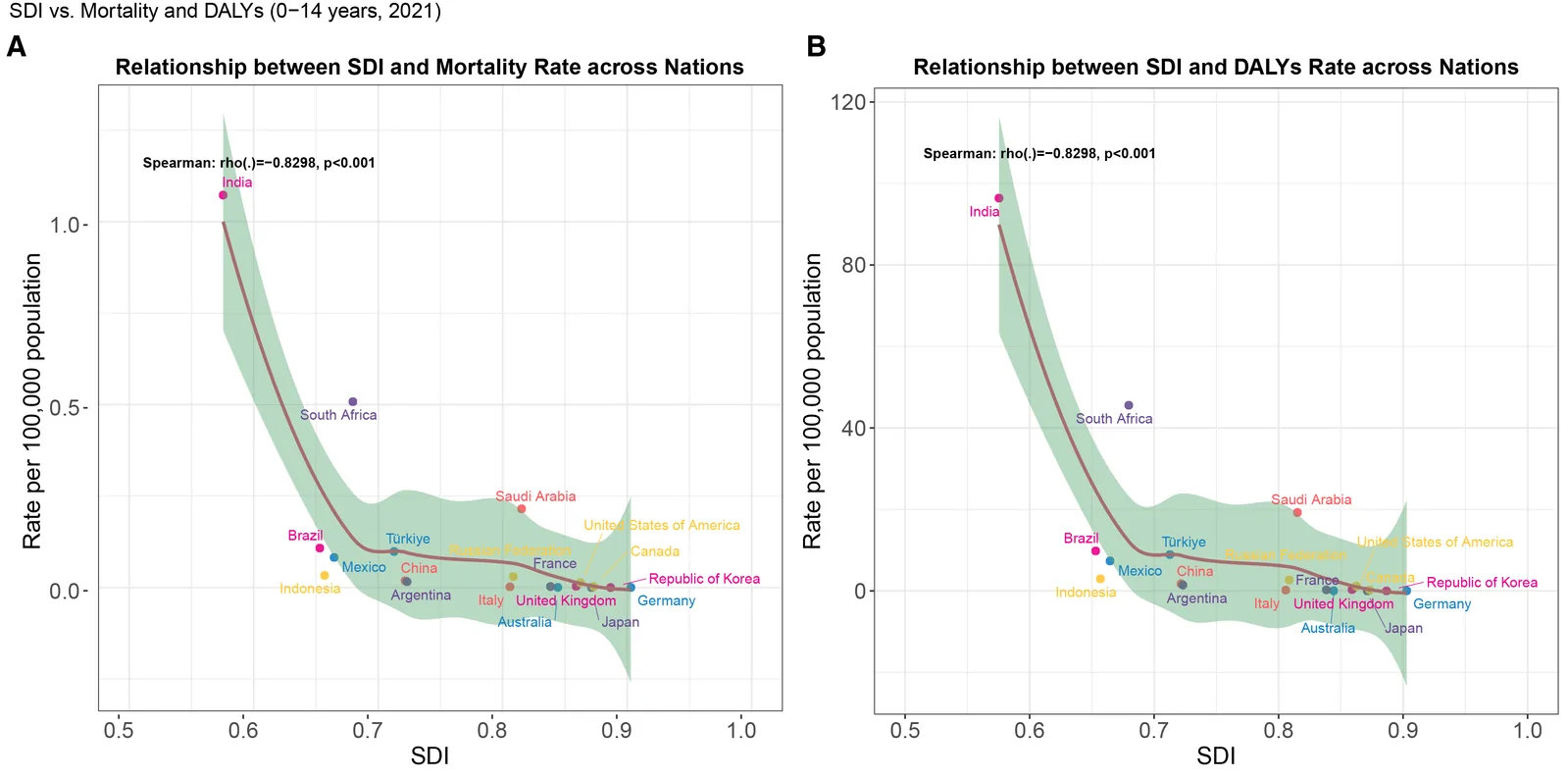
Correlation between the Sociodemographic Index (SDI) and the burden of RSV-associated lower respiratory tract infections in children aged 0–14 years in G20 countries, 2023 (A) Correlation of SDI with mortality rate. (B) Correlation of SDI with DALY rate. The curve represents an LOESS (locally estimated scatterplot smoothing) fit with a span parameter of 0.75, and the shaded band indicates the 95% pointwise confidence interval.

## Discussion

This study, based on the GBD 2023 database, analyzed the burden of pediatric RSV-associated lower respiratory tract infections in G20 countries from 1990 to 2023. The findings revealed that the number of deaths and DALYs decreased substantially from 45,619.852 to 4,091,464.436 in 1990 to 5,373.225 and 483,161.884 in 2023, respectively. During the COVID-19 pandemic period (2020–2021), RSV-related disease burden reached a historic nadir but rebounded sharply from the pandemic-era nadir in 2022–2023, though the 2023 rates remained well below pre-pandemic levels. Age-stratified analyses demonstrated that infants aged <1 year bore the greatest burden and exhibited a rapid declining rate. Substantial country-level disparities were observed, with India carrying the heaviest burden. Moreover, SDI showed a strong inverse correlation with disease burden across G20 nations. These findings may provide updated evidence to inform the optimization of pediatric RSV prevention and control strategies and the targeted use of vaccines and monoclonal antibodies in the post-pandemic era.

The present study observed an atypical fluctuation pattern of pediatric RSV-associated lower respiratory tract infection burden, characterized by a precipitous decline followed by a notable increase after 2019. Between 2020 and 2021, global RSV transmission fell to historic lows, a trend closely associated with the widespread implementation of non-pharmaceutical interventions.^4^ Measures such as mask mandates, closure of public venues, and travel restrictions during the COVID-19 pandemic profoundly disrupted RSV transmission chains, driving RSV detection rates to near-zero levels. This phenomenon was not unique to RSV. Multiple studies have shown that other respiratory pathogens, including influenza virus and parainfluenza virus, also experienced substantial declines in transmission during non-pharmaceutical interventions (NPI) implementation.^15^^,^^16^^,^^17^ However, unlike influenza and certain other viruses, RSV exhibited a more pronounced resurgence upon relaxation of NPIs. During 2022–2023, RSV activity in many countries and regions markedly exceeded pre-pandemic levels, a phenomenon termed “immune debt.”^8^^,^^9^ To quantify this descriptive increase within G20 countries, we compared mortality and DALY rates at three time points (Tables S2 and S3): the mortality rate declined by 69.0% from 2019 (1.395/100,000) to 2021 (0.432/100,000), then rebounded by 28.7% to 0.556/100,000 in 2023, with the most pronounced increases observed in Indonesia, Turkey, and Brazil, suggesting a substantial resurgence across G20 members following the relaxation of pandemic restrictions.

The notable increase in RSV activity during 2022–2023 carries important implications. First, epidemiological surveillance systems must maintain high sensitivity to enable early warnings of such changes. Second, clinicians should remain vigilant regarding potential changes in the clinical presentation of RSV infection, particularly rising trends in intensive care unit (ICU) admissions and the need for mechanical ventilation.

Our study found a marked age-specific distribution of pediatric RSV disease burden. Infants aged <1 year bore the highest burden, and younger age was associated with heavier burden. The propensity for severe RSV infection during early childhood may involve two key mechanisms. Anatomically, infants have narrow bronchial lumina and weak cartilaginous support, predisposing them to airway obstruction following mucous secretion. Immunologically, the infant immune system remains immature, with incompletely developed innate and adaptive immune responses.^18^^,^^19^

Based on established external evidence, certain infant groups should be prioritized for severe RSV screening and early intervention, particularly those aged <3 months, preterm and low-birth-weight infants, and those with underlying conditions such as congenital heart disease, chronic lung disease, or neuromuscular disorders, as these are well-documented high-risk factors for severe RSV infection.^9^ During peak RSV seasons, pediatric emergency departments and outpatient clinics should enhance active screening of these high-risk infants.

Our study also revealed marked heterogeneity in the burden of pediatric RSV-associated lower respiratory tract infections across G20 countries, with India, Indonesia, Mexico, Saudi Arabia, and South Africa carrying the highest burdens. Zhao et al.,^13^ using GBD 2021 data, reported that the global burden was concentrated in low-SDI regions, a pattern that is also reflected within the G20, where lower-SDI members bear disproportionately higher disease burdens. The mechanisms underlying these disparities are multidimensional. First, in lower-resource settings within the G20, such as parts of India, Indonesia, and South Africa, inadequate primary healthcare facilities, shortages of trained personnel, and limited respiratory support equipment likely prevent many children with RSV from receiving timely and effective treatment. Second, malnutrition may compromise children’s mucosal barrier function and immune responses. Third, high population density, along with air and environmental pollution, increases RSV transmission risk. From a policy perspective, priority interventions should focus on the highest-burden G20 countries, particularly India, Indonesia, Mexico, Saudi Arabia, and South Africa, while also addressing the needs of underserved populations within higher-income member states.

Spearman’s rank correlation analysis demonstrated a significant inverse relationship between SDI and pediatric RSV disease burden, consistent with previous findings using GBD 2019 data, which also reported that lower-SDI regions carry the highest RSV burden. The advantages of high-SDI regions include well-established immunization programs, good nutritional status, advanced medical equipment, and highly trained personnel. These factors collectively create a protective network that shields children from severe RSV infection.

Our findings have clear implications for the precise implementation of RSV prevention strategies. First, in high-SDI regions, where the RSV burden is already low, prevention efforts should focus on further reducing hospitalization rates, including promoting routine neonatal administration of nirsevimab and enhancing targeted prevention for high-risk infants. Studies have shown that long-acting monoclonal antibodies can achieve an overall effectiveness of 79% against RSV hospitalization in children aged <24 months.^20^ Second, in middle-SDI regions, prevention priorities should consolidate existing immunization programs while establishing robust RSV surveillance networks. Third, in low-SDI regions, there is an urgent need to strengthen primary health care systems, improve nutritional status, and seek affordable and convenient RSV prevention options.

In conclusion, from 1990 to 2023, the disease burden of pediatric RSV-associated lower respiratory tract infections declined substantially, with a descriptive increase observed from the 2021 nadir to 2023, although 2023 rates remained below pre-pandemic levels. The disease burden is highly concentrated in low-SDI regions and among children <1 year. There is a significant inverse correlation between pediatric RSV burden and SDI. Given the characteristics of age polarization and SDI correlation, RSV prevention and control efforts should adhere to the principle of precision stratification, prioritizing protection of the most vulnerable infant populations and the most resource-constrained regions.

### Limitations of the study

This study has several limitations. First, the GBD database estimates rely on modeling approaches, and the quality and completeness of source data vary considerably across regions. Especially in low- and middle-income countries, the health information registration systems in many of these nations are not well developed, which may lead to deviations in the estimation of disease burdens in these regions. The national-level Spearman correlation analysis among G20 countries did not adjust for potential non-independence of data points, which may slightly affect the precision of the estimates. However, the limited number and geographic diversity of G20 members reduce the clustering effect. Second, because the latest data in the GBD 2023 database cover up to 2023, this study could not further analyze potential additional rebounds or seasonal pattern changes in RSV during 2024–2025. Future research should incorporate updated data to validate whether the trends observed in this study persist. Third, this study focused primarily on mortality and DALY burden of RSV-associated lower respiratory tract infections and did not include the burden of outpatient or milder RSV infections, which are essential for comprehensively assessing the impact of RSV and formulating health policies. BAPC projections are statistical extrapolations based on pre-pandemic trends, not forecasts of interventions. Fourth, the overall EAPC masks the pandemic structural break; we therefore calculated segmented EAPCs, and the SDI analysis is ecological and descriptive, not causal. All secondary analyses used GBD point estimates, so uncertainty intervals are conditional on these estimates.

## Resource availability

### Lead contact

Requests for further information and resources should be directed to and will be fulfilled by the lead contact, Jie Zhang (897989652@qq.com).

### Materials availability

This study did not generate new unique reagents.

### Data and code availability

• All data used in this study are publicly available from the Global Burden of Disease 2023 database, accessible via the GBD Results Tool (http://ghdx.healthdata.org/gbd-results-tool). The G20 aggregate and country-level estimates were extracted directly from the tool.
• The custom R scripts used for data curation, EAPC calculations, and Spearman correlation analyses are available from the corresponding author upon reasonable request.
• Any additional information required to reanalyze the data reported in this paper is available from the lead contact upon request.

## Acknowledgments

This study was supported by the Yunnan Provincial Clinical Medicine Research Center for Gynecological and Obstetric Diseases (grant nos. 202505AJ310008 and 2026YJZX-FC22), the Xingdian Healthcare Talents Program (2025), Yunnan Provincial Ten-10.13039/501100010871Thousand Talents Program for Famous Medical Experts (grant no. KH-2025-XDYC-YLWS-07), and the Open Project of NHC Key Laboratory of Healthy Birth and Birth Defect Prevention in Western China (grant no. 2024XBYSKF018).

## Author contributions

Y.G. and Y.L. conducted data screening and quality evaluation. Y.G., J.Z., and Y.L. helped design study. Y.G. and Y.L. wrote the main manuscript text. Y.G., J.Z., and Y.L. made contributions to the revision of the paper. All authors revised, read, and approved the final manuscript.

## Declaration of interests

The authors declare no conflict of interests.

## STAR★Methods

### Key resources table

**Table:** 

REAGENT or RESOURCE	SOURCE	IDENTIFIER
**Data and software**		

GBD 2023 database	Institute for Health Metrics and Evaluation (IHME)	http://ghdx.healthdata.org/gbd-results-tool
R software (version 4.5.3)	R Foundation	https://www.r-project.org/
R packages: tidyverse, ggplot2, dplyr, stats	CRAN	https://cran.r-project.org/

### Experimental model and study participant details

This study involved no experimental models or human participants. All analyses were conducted on de-identified, aggregated secondary data from the GBD 2023 database.

### Method details

#### Data extraction

We extracted deaths and DALYs for RSV-associated lower respiratory tract infections in children aged 0–14 years in G20 countries from GBD 2023 (years 1990–2023). Extraction parameters: Etiology = “Respiratory syncytial virus”; Cause = “Lower respiratory infections”; Measure = “Deaths” and “DALYs”; Metric = “Number”, “Percent”, and “Rate”; Age = five subgroups (<1 year, 12–23 months, 2–4 years, 5–9 years, 10–14 years); Sex = Male, Female, Both. G20 aggregate estimates were extracted directly from the GBD Results Tool as the pre-defined “G20” location aggregate.

### Quantification and statistical analysis

All statistical analyses were performed using R version 4.5.3. Mortality and DALY rates per 100,000 children were calculated with 95% uncertainty intervals (UIs). Temporal trends were assessed using estimated annual percentage change (EAPC), calculated via log-linear regression: log(rate) = α + β × year + ε, where EAPC = 100 × (eˆβ − 1), with rates weighted by inverse variance. The 95% CIs for EAPC were derived from the standard error of β. For the post-2019 period, segmented EAPCs were calculated for 1990–2019, 2019–2021, and 2021–2023 as conditional point estimates; wide CIs reflect limited observations and should be interpreted cautiously. Spearman’s rank correlation was used to examine the association between SDI and disease burden at the country level (*n* = 19, 2023 cross-section), with ρ and P-values reported. Rate change from 1990 to 2023 was calculated as (Rate2023 − Rate1990)/Rate1990, with 95% UIs from GBD draws. All tests were two-sided; *p* < 0.05 was considered statistically significant. The EAPC and Spearman analyses used GBD point estimates rather than matched draws.

## Supplemental information

Table S1. Age-stratified mortality burden of RSV-associated lower respiratory tract infections in children aged 0–14 years across G20 countries, 1990 and 2023 (A) Mortality number and rate by age subgroup. (B) Rate change and EAPC by age subgroup.
