# Supplementary material for: Pediatric RSV-associated lower respiratory tract infection burden in G20 countries in 2023
Source: iScience. 2026 Oct 7;29(11):117788. doi: 10.1016/j.isci.2026.117788 (PMC13676714; doi:10.1016/j.isci.2026.117788)
Supplement: Supplementary file 1 [file mmc2.pdf]

Supplementary Table 2. Mortality burden of RSV-associated lower respiratory tract infections in children aged 0–14 years in G20 countries, 2019, 2021, and 2023.

location_name	2019		2021		2023	
	**Number (95 %UI)**	**Rate per 100,000(95%UI)**	**Number (95 %UI)**	**Rate per 100,000(95%UI)**	**Number (95 %UI)**	**Rate per 100,000(95%UI)**
G20	13933.141(12383.106-15434.629)	1.395(1.240-1.545)	4265.789(2080.902-7545.543)	0.432(0.211-0.763)	5373.225(3715.553-7862.622)	0.556(0.385-0.814)
Argentina	40.610(36.951-45.315)	0.379(0.344-0.422)	1.654(0.708-3.331)	0.016(0.007-0.032)	4.378(1.682-9.000)	0.043(0.017-0.089)
Australia	4.024(3.315-4.744)	0.086(0.071-0.101)	0.022(0.006-0.051)	0.000(0.000-0.001)	0.197(0.089-0.340)	0.004(0.002-0.007)
Brazil	300.430(266.716-331.717)	0.675(0.599-0.745)	47.393(13.101-108.437)	0.108(0.030-0.247)	116.388(55.308-208.382)	0.269(0.128-0.481)
Canada	3.957(3.371-4.615)	0.064(0.055-0.075)	0.163(0.041-0.404)	0.003(0.001-0.006)	3.213(2.081-4.453)	0.052(0.034-0.072)
China	541.169(466.253-617.020)	0.210(0.181-0.239)	48.582(25.565-85.599)	0.019(0.010-0.033)	130.378(71.719-202.861)	0.052(0.028-0.080)
United Kingdom	10.774(8.937-12.305)	0.107(0.089-0.122)	0.357(0.177-0.632)	0.004(0.002-0.006)	5.286(4.013-7.202)	0.053(0.040-0.072)
European Union	82.619(72.884-93.432)	0.120(0.106-0.136)	1.899(1.334-2.660)	0.003(0.002-0.004)	52.353(40.376-64.894)	0.078(0.060-0.096)
France	6.263(5.136-7.392)	0.051(0.042-0.060)	0.341(0.130-0.732)	0.003(0.001-0.006)	4.425(2.697-6.828)	0.036(0.022-0.056)
Germany	5.050(4.200-5.861)	0.044(0.037-0.051)	0.034(0.012-0.080)	0.000(0.000-0.001)	1.951(1.326-2.818)	0.016(0.011-0.024)
India	10741.170(9399.539-12048.175)	2.838(2.484-3.183)	3985.146(1754.828-7222.154)	1.073(0.472-1.944)	3908.194(2230.668-6327.152)	1.078(0.615-1.745)

Indonesia	854.070(612.044-1183.264)	1.247(0.893-1.727)	22.402(6.686-48.106)	0.033(0.010-0.071)	580.028(291.696-954.189)	0.866(0.435-1.425)
Italy	4.981(4.083-5.859)	0.063(0.052-0.074)	0.159(0.078-0.281)	0.002(0.001-0.004)	2.446(1.802-3.249)	0.033(0.025-0.044)
Japan	11.700(9.973-13.579)	0.076(0.065-0.088)	0.011(0.001-0.062)	0.000(0.000-0.000)	3.623(2.109-5.599)	0.025(0.015-0.039)
Mexico	389.687(353.856-434.846)	1.181(1.073-1.318)	26.384(12.047-47.792)	0.082(0.038-0.149)	298.051(204.084-410.774)	0.964(0.660-1.328)
Republic of Korea	2.213(1.892-2.588)	0.034(0.029-0.040)	0.000(0.000-0.000)	0.000(0.000-0.000)	0.787(0.499-1.115)	0.014(0.009-0.020)
Russian Federation	59.489(51.092-67.857)	0.244(0.210-0.279)	7.163(1.823-16.453)	0.030(0.008-0.069)	49.119(33.094-66.025)	0.213(0.144-0.286)
Saudi Arabia	85.497(70.849-104.159)	1.065(0.882-1.297)	17.446(7.971-32.421)	0.215(0.098-0.400)	62.446(30.335-105.212)	0.775(0.376-1.306)
South Africa	656.714(557.260-761.791)	4.115(3.492-4.773)	81.801(33.814-161.786)	0.508(0.210-1.005)	76.092(30.755-149.224)	0.473(0.191-0.927)
Türkiye	130.894(110.372-155.159)	0.683(0.576-0.810)	18.630(8.384-35.543)	0.098(0.044-0.188)	55.294(27.983-91.802)	0.299(0.151-0.496)
United States of America	82.932(72.231-95.126)	0.135(0.117-0.155)	8.053(3.653-14.828)	0.013(0.006-0.024)	70.194(49.111-90.272)	0.117(0.082-0.151)

Supplementary Table 3. DALY burden of RSV-associated lower respiratory tract infections in children aged 0–14 years in G20 countries, 2019, 2021, and 2023.

location_name	2019		2021		2023	
	**Number (95 %UI)**	**Rate per 100,000(95%U**	**Number (95 %UI)**	**Rate per 100,000(95%U**	**Number (95 %UI)**	**Rate per 100,000(95%U**

		I)		I)		I)
location_name	Value_2019	Value_2019	Value_2021	Value_2021	Value_2023	Value_2023
G20	1250255.284(1111611.295-1384066.986)	125.188(111.306-138.587)	383553.846(187260.317-678110.499)	38.800(18.943-68.596)	483161.884(334432.415-707829.900)	50.020(34.622-73.279)
Argentina	3641.140(3317.765-4055.833)	33.943(30.929-37.809)	149.064(63.590-298.782)	1.425(0.608-2.857)	393.724(151.592-807.626)	3.906(1.504-8.012)
Australia	363.008(301.362-427.312)	7.743(6.428-9.115)	2.040(0.515-4.584)	0.043(0.011-0.096)	17.967(8.210-30.945)	0.371(0.169-0.639)
Brazil	27055.637(24109.822-29790.253)	60.796(54.176-66.940)	4309.699(1186.276-9845.596)	9.805(2.699-22.400)	10492.359(5002.945-18688.355)	24.206(11.542-43.115)
Canada	356.418(304.996-414.529)	5.770(4.938-6.711)	14.835(3.726-36.483)	0.239(0.060-0.587)	289.449(187.602-400.950)	4.686(3.037-6.491)
China	49234.064(42371.251-55983.355)	19.070(16.412-21.684)	4537.008(2393.956-7901.104)	1.748(0.922-3.044)	12039.571(6592.956-18465.178)	4.776(2.615-7.325)
United Kingdom	971.854(809.453-1109.114)	9.627(8.018-10.987)	32.431(16.036-57.432)	0.321(0.159-0.568)	478.749(364.227-651.153)	4.772(3.630-6.490)
European Union	7519.800(6637.321-8455.110)	10.964(9.677-12.327)	174.717(122.971-244.046)	0.256(0.180-0.358)	4772.517(3683.776-5907.908)	7.075(5.461-8.758)
France	573.480(471.649-671.655)	4.678(3.847-5.479)	31.526(12.052-67.868)	0.258(0.098-0.554)	405.184(244.599-618.758)	3.340(2.016-5.100)
Germany	464.705(389.917-535.717)	4.073(3.418-4.696)	3.235(1.116-7.610)	0.028(0.010-0.065)	185.270(125.478-265.274)	1.545(1.047-2.213)
India	963455.202(843604.737-1079744.309)	254.565(222.898-285.291)	358132.420(157693.978-648822.216)	96.401(42.448-174.649)	351293.485(200755.870-568712.054)	96.875(55.362-156.832)
Indonesia	76499.823(54750.735-105922.884)	111.650(79.908-154.593)	2010.196(604.581-4312.984)	2.966(0.892-6.363)	52156.497(26307.863-85669.268)	77.865(39.275-127.897)
Italy	452.874(373.808-532.513)	5.746(4.743-6.756)	14.587(7.098-25.814)	0.191(0.093-0.339)	223.349(164.873-293.647)	3.040(2.244-3.997)

Japan	1061.929(910.635-1228.787)	6.871(5.892-7.951)	1.011(0.050-5.727)	0.007(0.000-0.038)	335.552(197.034-518.450)	2.316(1.360-3.578)
Mexico	34794.788(31595.981-38813.587)	105.493(95.795-117.678)	2354.973(1076.563-4268.094)	7.348(3.359-13.318)	26582.415(18204.785-36629.842)	85.970(58.876-118.464)
Republic of Korea	204.244(174.364-235.510)	3.138(2.679-3.618)	0.025(0.019-0.033)	0.000(0.000-0.001)	74.458(47.778-106.103)	1.310(0.841-1.867)
Russian Federation	5351.144(4605.148-6088.103)	21.983(18.918-25.010)	644.427(163.985-1479.382)	2.706(0.689-6.213)	4412.833(2973.744-5935.513)	19.148(12.904-25.755)
Saudi Arabia	7632.796(6318.640-9292.099)	95.037(78.674-115.697)	1561.108(714.116-2899.373)	19.249(8.805-35.751)	5581.475(2709.389-9390.747)	69.261(33.621-116.530)
South Africa	58804.085(49878.364-68228.790)	368.467(312.538-427.522)	7337.835(3033.557-14523.276)	45.571(18.840-90.196)	6818.674(2761.872-13367.169)	42.370(17.162-83.062)
Türkiye	11754.057(9934.783-13913.185)	61.345(51.851-72.614)	1683.873(758.822-3198.972)	8.897(4.009-16.902)	4997.132(2541.879-8265.989)	27.022(13.745-44.699)
United States of America	7447.098(6527.440-8522.000)	12.097(10.604-13.844)	729.119(329.544-1343.884)	1.202(0.543-2.215)	6316.961(4402.445-8102.637)	10.570(7.367-13.558)
